# Liver-Specific γ-Glutamyl Carboxylase-Deficient Mice Display Bleeding Diathesis and Short Life Span

**DOI:** 10.1371/journal.pone.0088643

**Published:** 2014-02-10

**Authors:** Kotaro Azuma, Tohru Tsukui, Kazuhiro Ikeda, Sachiko Shiba, Kimie Nakagawa, Toshio Okano, Tomohiko Urano, Kuniko Horie-Inoue, Yasuyoshi Ouchi, Masahito Ikawa, Satoshi Inoue

**Affiliations:** 1 Department of Geriatric Medicine, Graduate School of Medicine, The University of Tokyo, Bunkyo-ku, Tokyo, Japan; 2 Division of Gene Regulation and Signal Transduction, Research Center for Genomic Medicine, Saitama Medical University, Hidaka, Saitama, Japan; 3 Department of Hygienic Sciences, Kobe Pharmaceutical University, Kobe, Hyogo, Japan; 4 Department of Anti-Aging Medicine, Graduate School of Medicine, The University of Tokyo, Bunkyo-ku, Tokyo, Japan; 5 Research Institute for Microbial Diseases, Osaka University, Suita, Osaka, Japan; Institut National de la Santé et de la Recherche Médicale, France

## Abstract

Vitamin K is a fat-soluble vitamin that plays important roles in blood coagulation and bone metabolism. One of its functions is as a co-factor for γ-glutamyl carboxylase (Ggcx). Conventional knockout of *Ggcx* causes death shortly after birth in homozygous mice. We created *Ggcx*-floxed mice by inserting *loxP* sequences at the sites flanking exon 6 of *Ggcx*. By mating these mice with albumin-Cre mice, we generated Ggcx-deficient mice specifically in hepatocytes (*Ggcx^Δliver/Δliver^* mice). In contrast to conventional *Ggcx* knockout mice, *Ggcx^Δliver/Δliver^* mice had very low activity of Ggcx in the liver and survived several weeks after birth. Furthermore, compared with heterozygous mice (*Ggcx^+/Δliver^*), *Ggcx^Δliver/Δliver^* mice had shorter life spans. *Ggcx^Δliver/Δliver^* mice displayed bleeding diathesis, which was accompanied by decreased activity of coagulation factors II and IX. *Ggcx*-floxed mice can prove useful in examining Ggcx functions *in vivo*.

## Introduction

Vitamin K is a fat-soluble vitamin, which is involved in blood coagulation and bone metabolism. One of the major functions of vitamin K is its role as a co-factor for γ-glutamyl carboxylase (Ggcx) [1]. Ggcx is responsible for the posttranslational modification of glutamic acid (Glu) residues into γ-carboxyglutamic acid (Gla) by its carboxylase activity. Hitherto, 19 kinds of Gla proteins have been found, that is, coagulation factors II, VII, IX, and X; protein C, protein S, and protein Z [2]; osteocalcin [3]; nephrocalcin [4]; matrix Gla protein [5]; growth arrest specific-6 (Gas6) [6]; periostin [7]; βIg-H3 [7]; proline-rich Gla protein 1 and 2 [8]; transmembrane Gla protein 3 and 4 [9]; upper zone of growth plate and cartilage matrix associated protein (UCMA; also called Gla-rich protein, GRP) [10]; and Ggcx itself, which was also shown to be γ-carboxylated [11]. Considering the various expression sites and functions of these Gla proteins, it is indicated that vitamin K is involved in many physiological and pathological processes by activating Ggcx.

On the other hand, we have previously demonstrated γ-carboxylation-independent vitamin K function, in which vitamin K is involved in the transcriptional regulation of nuclear receptor SXR/PXR [12]. We reported that SXR/PXR-dependent vitamin K functions are actually involved in the biological process in osteoblasts [13] and hepatocellular carcinoma cells [14].

To fully understand the function of vitamin K, it is vital to separate Ggcx-dependent and SXR/PXR-dependent vitamin K functions. Analysis of *Ggcx* knockout mice would be useful in examining Ggcx-dependent vitamin K functions in each tissue; however, this attempt has been hampered by the fact that *Ggcx* knockout mice die between embryonic day 9.5 and 18, and the few that survive to term die shortly after birth [15]. To overcome this limitation, we generated *Ggcx*-floxed mice that enabled organ specific deficiency of Ggcx when bred with transgenic Cre mice that showed organ-specific expression of Cre recombinase. Here, we report a phenotype with liver-specific deficiency of Ggcx.

## Materials And Methods

### Ethics Statement

This study was carried out in strict accordance with the consent of the Animal Care and Use Committees of Osaka University and Saitama Medical University. The protocol was approved by the Committee on the Ethics of Animal Experiments of the University of Osaka (Protocol Number: 2003G004) and Saitama Medical University (Protocol Number: 797).

### Targeting Vector Construction

A targeting vector was constructed using pNT1.1 containing two *loxP* sequences, a phosphoglycerate kinase (pgk)-neomycin selectable marker cassette (neomycin cassette), and a herpes simplex virus thymidine kinase gene. A mouse 129 strain λ genomic library (Stratagene) was purchased and digested with NotI. Genomic fragments were subcloned into pBluescript SK(+). The 5′ homology arm of the construct was derived from an Asp718/HindIII genomic fragment containing intron 4, exon 5, and intron 5 of *Ggcx*. This fragment was subcloned into pBluescript SK(+) and then inserted into the 5′ region of pNT1.1 between the NotI and SalI sites. The 3′ homology arm of the construct was derived from a genomic fragment containing intron 6, exon 7, and intron 7 of *Ggcx*. This fragment was amplified with primers 5′-GCTTAATTAAATGCATATAAGACAAGCACC-3′ and 5′-ATGGTACCTAGGAAAGCAGGAAGAAG-3′ and inserted into the 3′ region of pNT1.1 at the PacI and Asp718 sites. The genomic region containing exon 6 was amplified with primers 5′-AAGCTTGCAGGTGATTCTCC-3′ and 5′-ATGCATAAAACAGAAAAAGTGAGCAAGCC-3′; it was then inserted into pNT1.1 at a BamHI site between the 5′-*loxP* site and neomycin cassette. This resulted in a targeting vector with a neomycin cassette between exon 6 and 7 and a thymidine kinase gene located downstream of the 3′ homology region. The targeting construct was linearized with NotI and electroporated into D3 ES cells [16].

### Generation of *Ggcx^flox/flox^* mice

Colonies of ES cells carrying the recombinant allele were screened using 150 µg/ml of G418 and negatively selected using 2 µM gancyclovir. Selected cells were amplified and genomic DNA was screened by Southern blot analysis. The ES cell lines carrying the recombinant allele were subsequently used to generate chimeras by injection into 129/Sv blastocysts. The chimeric mice were mated with wild type C57BL/6N mice. The F_1_ agouti offspring were analyzed for homologous recombination by Southern blotting and PCR analysis. The F_1_ offspring were backcrossed to C57BL/6N mice for more than eight generations to generate *Ggcx^flox/+^* mice with a C57BL/6N genetic background. *Ggcx^flox/+^* mice were intercrossed to generate *Ggcx^flox/flox^* mice containing homozygous recombinant alleles.

### Generation of hepatocyte-specific Ggcx-deficient mice

C57BL6/J mice containing transgenic constructs of mouse albumin enhancer/promoter and Cre recombinase modified to include a nuclear localization sequence (Alb-Cre) were purchased from the Jackson Laboratory. ROSA26-LacZ reporter mice were also obtained from the Jackson Laboratory. Hepatocyte-specific expression of Cre recombinase was confirmed by mating Alb-Cre mice with ROSA26-LacZ mice and assessing the β-galactosidase activity of the expressed *LacZ* gene, which is expected to be detected in cells expressing functional Cre recombinase. To generate hepatocyte-specific Ggcx-deficient mice (*Ggcx^Δliver/Δliver^*), Alb-Cre mice were mated with *Ggcx^flox/flox^* mice and F_1_ offspring were subsequently intercrossed.

### Southern blotting

EcoRI digested genomic DNA—derived from ES cells or tail specimens—was electrophoresed through a 0.6% agarose gel, transferred to a Hybond N+ membrane (Amersham Bioscience), and hybridized with the ^32^P-labeled 164-bp sequence (ACAGCTTTCTTGATGCTGCTGGACATTCCCCAGGAACGCGGCCTTAGCTCCCTGGACCGAAAATACTTGGATGGGCTGGATGTGTGCCGTTTCCCCTTGCTGGATGCCTTGCGCCCACTGCCACTGGACTGGATGTATCTTGTCTACACCATCATGTTTCTGGG) in exon 3 of the *Ggcx* gene.

### Genotyping

Genomic DNA derived from tail specimens was used as the template for PCR analysis. Tail cut was done before 3 weeks old or immediately after the mice died. The Cre recombinase gene was detected by amplifying a 654-bp fragment within the *Cre* gene with primers 5′-CCTGGAAAATGCTTCTGTCCGTTTGCC-3′ and 5′-GAGTTGATAGCTGGCTGGTGGCAGATG-3′. The *loxP* sequence was detected by amplifying the *Ggcx* sequence with primers 5′-AACTTAGGGAGTTGGTTCTCTTTCACTT-3′ and 5′-AATCCAATACACCCAAGGTCTTATTCAT-3′ in intron 5, containing *loxP* and linker sequences, to yield a 454-bp fragment from the *loxP*-containing allele and 407-bp fragment from the wild type allele. Deletion of exon 6 in the liver was confirmed with primers 5′- CGTGTACTTCATCGCGGGTG-3′ within exon 6 and 5′-TCTGTATCCGGCTGAACGGG-3′ within intron 6. DNA samples derived from liver, spleen, kidney and heart of both *Ggcx^Δliver/Δliver^* mice and control *Ggcx^+/+^* mice. The DNA samples of same concentration (3 ng/µl) were used as templates for PCR analysis.

### Animal experiments

Mice were housed in a temperature-controlled room (22°C) with a 12-h light/dark schedule, had free access to water, and were fed standard laboratory chow. When mice were sacrificed, anesthesia with an intraperitoneal injection of 2.5% avertin was employed to minimize suffering of animals. Exsanguination was done following anesthesia to ensure death.

### Ggcx activity assay

FLEEL was purchased from Bachem (Philadelphia, PA). Lα-phosphatidylcholine (type VE) and CHAPS were obtained from Sigma Aldrich Japan (Tokyo, Japan). Vitamin K_2_ (menaquinone-4) was obtained from Eisai Co., Ltd. (Tokyo, Japan). The peptide ProFIX19, which contains the sequence AVFLDHENANKILNRPKRY, was synthesized by Genenet Co., Ltd. (Fukuoka, Japan). NaH^14^CO_3_ (specific activity, 58 mCi/mmol) was obtained from Amersham Biosciences Corp. (NJ).

Six-week old mice were anesthetized with an intraperitoneal injection of 2.5% avertin and the livers were excised for measurement of Ggcx activity. Mice were euthanized by exsanguination following liver excision. The Ggcx activity was measured as previously described [17]. The amount of ^14^CO_2_ incorporated into exogenous substrates was measured in reaction mixtures of 125 µl containing substrate (3.6 mM FLEEL), 222 µM reduced vitamin K (vitamin KH_2_), 16 µM propeptide ProFIX19, 1.4 mM NaH^14^CO_3_ (5 µCi), 25 mM MOPS (pH 7.0), 500 mM NaCl, 0.16% (w/v) phosphatidylcholine, 0.16% (w/v) CHAPS, 8 mM DTT, and 0.8 M ammonium sulfate, unless stated otherwise. All of the assay components, except for the microsomal fraction, were prepared as master mixes. ^14^CO_2_ incorporation into peptide substrates (after an incubation period of over 30 min) was assayed using a scintillation counter. All assays were performed in quadruplicate.

### Coagulation factor activity assay

Blood was collected from 6-week-old mice under anesthesia with an intraperitoneal injection of 2.5% avertin. Collected blood was immediately combined with one-tenth volume of 110 mM sodium citrate. Plasma was isolated by centrifugation for 15 min at 2500×*g*. The obtained plasma was analyzed with an automated blood coagulation analyzer (STA Compact, Roche, Basel, Switzerland) to determine factor II and IX activity using prothrombin (factor II) or factor IX-deficient plasma.

### Bleeding test

Four-week-old mice were anesthetized with an intraperitoneal injection of 2.5% avertin. Their tails were cut to yield the same wound diameters. To evaluate bleeding time, filter paper was applied to the edge of the wound every minute, taking care not to dislodge the clot.

### Hematological examination

Two ml of blood was collected from 6-week-old mice under anesthesia with an intraperitoneal injection of 2.5% avertin. Collected blood was mixed with anti-coagulants (1 mg of EDTA-2K and 20 µl of 3% EDTA-3K). The number of platelets was measured using the Advia 120 (Bayer, Dublin, Ireland).

### Life span analysis

To evaluate lifespan, mice were kept with their littermates. Male and female mice were kept in separate cages without mating. They were kept until either natural death, or evidence of impending mortality necessitating euthanasia, such as unresponsiveness to touch, slow respiration, coldness to touch, a hunched up position with matted fur. Condition of the mice was monitored every two days.

### Statistical analysis

Data are expressed as mean ± SEM. Differences between the mean values were analyzed using the unpaired Student's *t*-test. Survival rates were plotted using the Kaplan-Meier method. Survival differences between the groups were analyzed using the log-rank test, for which p-values were adjusted by the Bonferroni method.

## Results

### Generation of hepatocyte-specific Ggcx-deficient mice

**Figure 1:**
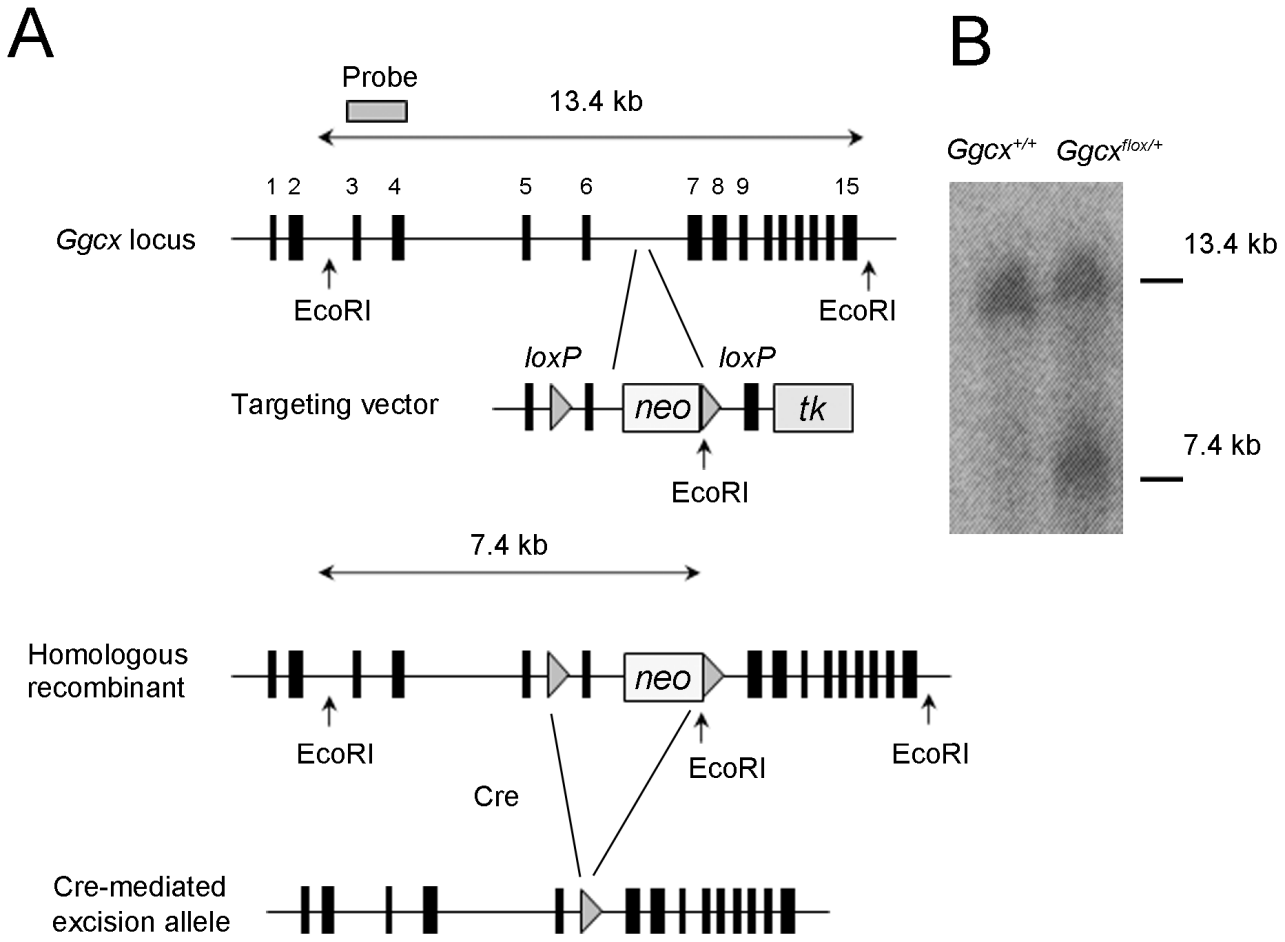
Cre-recombinase-mediated tissue-specific excision of *Ggcx*. A, Strategy for homologous recombination. Targeting vector was designed to flank exon 6 of the *Ggcx* gene, and frame shift was generated by excision with Cre recombinase. Two *loxP* sequences (triangles) were inserted into introns 5 and 6. Neomycin cassette and EcoRI site were inserted into intron 6. B, Southern blot analysis of tail DNA. Homologous recombinant allele generated a 7.4-kb fragment by EcoRI digestion. A representative figure is shown.

**Figure 2:**
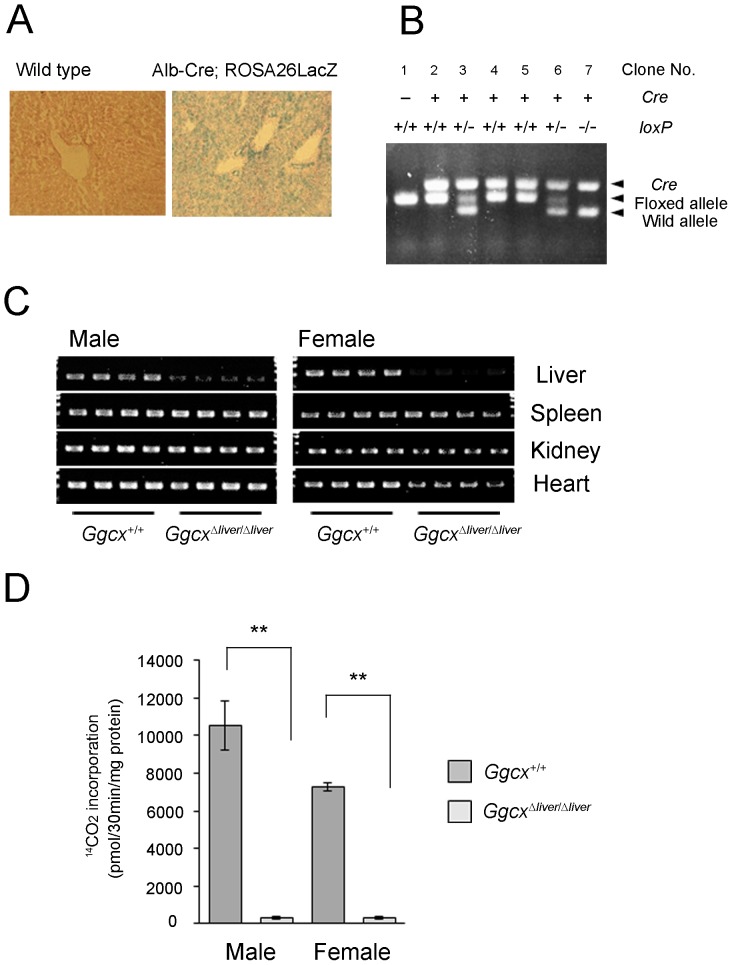
Liver-specific ablation of *Ggcx* gene. A, The albumin promoter is active only in hepatocytes. Alb-Cre or wild type (WT) mice were mated with ROSA26-LacZ mice. Livers were obtained postnatally from mice and stained with X-gal. β-galactosidase activity was detected in the hepatocytes of the mouse born from mating of Alb-Cre and ROSA26-LacZ mice (right panel). Liver of the mouse born from wild type was shown as a negative control (left panel). Representative figures are shown for each group. B, Genotyping using genomic tail DNA. Expected bands from Cre recombinase, *loxP* sequence (floxed allele), and wild type allele are shown. In mouse #2, #4 and #5, both alleles were replaced with *loxP* containing recombinant alleles, with at least one copy of Cre recombinase. A representative result is shown. C, Liver-specific ablation of *Ggcx* gene was confirmed with PCR analysis. DNA samples derived from liver, spleen, kidney and heart of 6-month week old *Ggcx^Δliver/Δliver^* mice (4 male mice and 4 female mice) and control *Ggcx^+/+^* mice (4 male mice and 4 female mice) were used as templates. D, Activity of Ggcx in the liver-specific Ggcx-deficient mice. Microsome was prepared from the livers of 6-week old *Ggcx^Δliver/Δliver^* mice and control *Ggcx^+/+^* mice of both sexes. The activity of Ggcx was measured by ^14^CO_2_ incorporated into exogenous substrate in the presence of reduced vitamin K (222 µM). Bars represent the mean value ± SEM (n = 4). Differences between the mean values were analyzed using the unpaired Student's *t*-test. **P<0.01.

The mouse γ-glutamyl carboxylase (*Ggcx*) gene consists of 15 exons (Figure 1A). To disrupt the *Ggcx* gene, the targeting vector was designed to flank exon 6 with two *loxP* sequences, and a frameshift was generated by excision with Cre recombinase (Figure 1A). Insertion of *loxP* sequences by homologous recombination was confirmed with Southern blotting analysis (Figure 1B). To delete the *Ggcx* gene in the liver alone, albumin-Cre (Alb-Cre) transgenic mice were used. The cre recombinase gene is under the control of the albumin promoter, which is active only in hepatocytes from E16.5 embryos [18] and the full activity was exhibited at 2 months after birth [19]. To confirm the specificity of recombination, the Alb-Cre mice were crossed with ROSA26-LacZ mice, which contain a reporter gene in which β-galactosidase is expressed in any tissue, and expression is dependent on Cre-mediated recombination. β-galactosidase activity was detected in the hepatocytes of offspring born from the mating of Alb-Cre with ROSA26-LacZ mice (Figure 2A); these offspring also showed Cre-recombinase activity in hepatocytes. Alb-Cre mice were then mated with *Ggcx*-floxed mice (*Ggcx^flox/flox^*) and the resulting F_1_ offspring were intercrossed. To examine the genotypes of the F_2_ offspring, the Cre recombinase gene and the *loxP*-containing region of the *Ggcx* gene were amplified by PCR using genomic DNA prepared from tail samples. Some mice that expressed Cre recombinase and carried homozygous floxed alleles were considered to be liver-specific Ggcx-deficient mice (*Ggcx^Δliver/Δliver^* mice) (Figure 2B). They were born alive and survived for at least several weeks. To confirm the ablation of *Ggcx* in the livers of *Ggcx^Δliver/Δliver^* mice, genomic DNA was extracted from liver and other organs (spleen, kidney and heart) from 6-week old *Ggcx^Δliver/Δliver^* mice and control *Ggcx^+/+^* mice. Decreased intensity of PCR products from exon 6 was observed in only livers of *Ggcx^Δliver/Δliver^* mice (Figure 2C). Next, vitamin K-dependent Ggcx activity was measured in the livers of 6-week old *Ggcx^Δliver/Δliver^* mice and control littermates (*Ggcx^+/+^* mice). Ggcx activity was significantly decreased in the livers of *Ggcx^Δliver/Δliver^* mice (Figure 2D). There was no significant difference in Ggcx activity between male and female *Ggcx^Δliver/Δliver^* mice.

### Bleeding diathesis in *Ggcx^Δliver/Δliver^* mice

**Figure 3:**
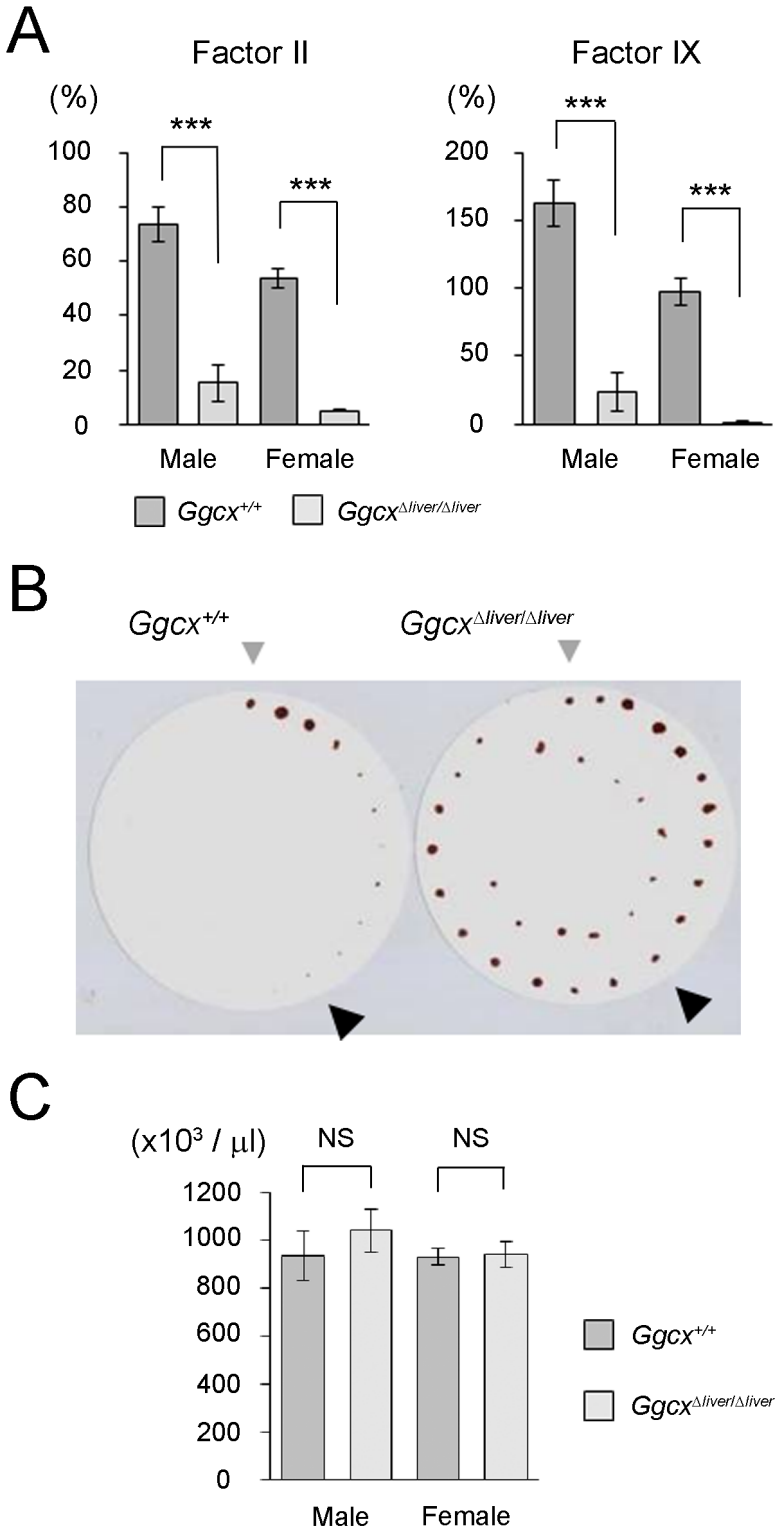
Bleeding diathesis in *Ggcx^Δliver/Δliver^* mice. A, Decreased activity of coagulation factor in *Ggcx^Δliver/Δliver^* mice. Activities of factors II and IX were significantly decreased in *Ggcx^Δliver/Δliver^* mice compared with wild type (*Ggcx^+/+^*) mice. Bars represent the mean value ± SEM (n = 8). Differences between the mean values were analyzed using the unpaired Student's *t*-test. ***P<0.001. B, Prolonged bleeding time in *Ggcx^Δliver/Δliver^* mice. Tail bleeding time was measured by a filter paper method. *Ggcx^Δliver/Δliver^* mice continued bleeding for more than 30 min. Gray triangle: 0 min; Black triangle: 10 min. A representative figure is shown. C. Platelet counts of *Ggcx^Δliver/Δliver^* mice. Hematological examination of male *Ggcx^Δliver/Δliver^* mice (n = 6), female *Ggcx^Δliver/Δliver^* mice (n = 12), male wild type mice (n = 10), and female wild type mice (n = 10) was performed. The platelet count was not significantly different between wild type mice and *Ggcx^Δliver/Δliver^* mice. Bars represent the mean value ± SEM. NS: not significant.

To examine the effect of decreased Ggcx activity in the livers of *Ggcx^Δliver/Δliver^* mice, the activities of vitamin K-dependent coagulation factors were examined. Activities of factors II and IX were significantly decreased in *Ggcx^Δliver/Δliver^* mice, compared with control (*Ggcx^+/+^*) mice (Figure 3A). Decreased activity of vitamin K-dependent coagulation factor caused bleeding diathesis in *Ggcx^Δliver/Δliver^* mice. Wild-type mice ceased bleeding within 10 minutes of tail incision, while *Ggcx^Δliver/Δliver^* mice continued to bleed for more than 30 minutes (Figure 3B). The platelet count was not significantly different between wild type mice and *Ggcx^Δliver/Δliver^* mice (Figure 3C), suggesting the longer bleeding time in *Ggcx^Δliver/Δliver^* mice was due to defective secondary coagulation.

### Shorter life span of *Ggcx^Δliver/Δliver^* mice

**Figure 4:**
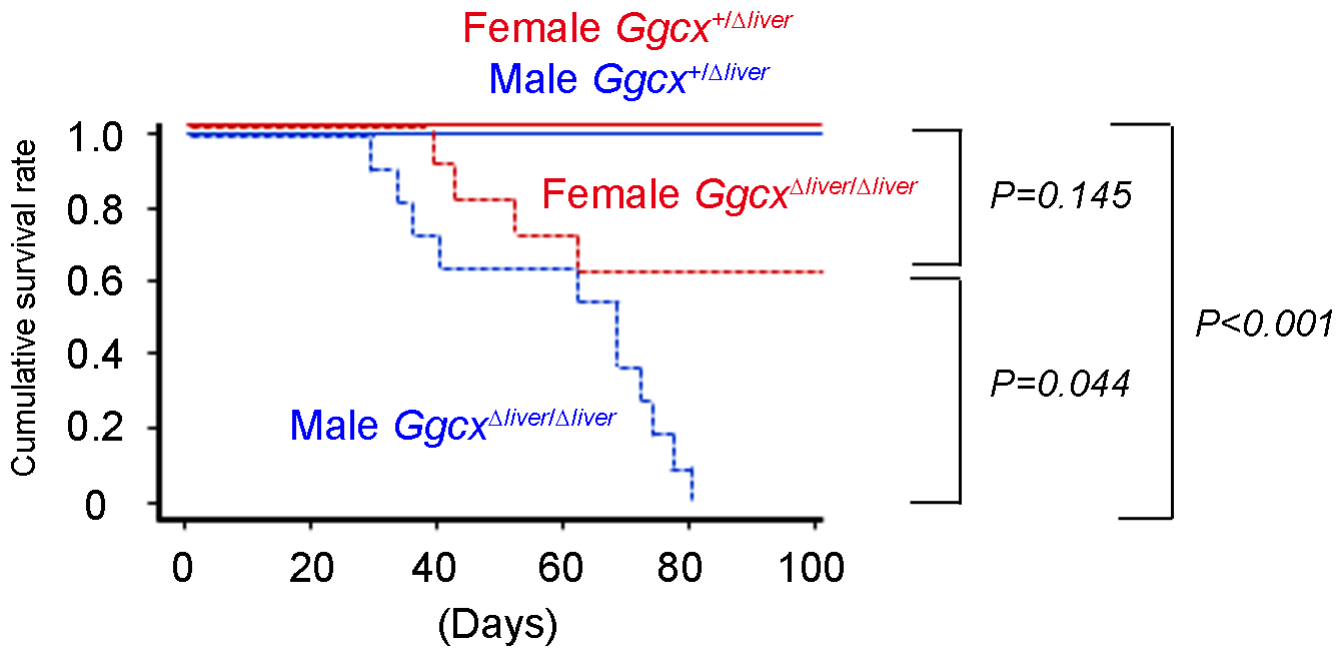
Shorter life span of *Ggcx^Δliver/Δliver^* mice. Cumulative life spans of male *Ggcx^Δliver/Δliver^* mice (n = 10), female *Ggcx^Δliver/Δliver^* mice (n = 11), male heterozygous littermates (*Ggcx^+/Δliver^* mice) (n = 12), and female heterozygous littermates (*Ggcx^+/Δliver^* mice) (n = 12) were calculated by the Kaplan-Meier method and compared using the log-rank test. P-values were adjusted by the Bonferroni method. Life span of male *Ggcx^Δliver/Δliver^* mice was significantly shorter (*P*<0.001) compared with that of heterozygous mice. The life spans of female *Ggcx^Δliver/Δliver^* mice were significantly longer than those of male *Ggcx^Δliver/Δliver^* mice (*P* = 0.044).

As a result of bleeding diathesis, injury and pregnancy caused fatal bleeding in *Ggcx^Δliver/Δliver^* mice. In 9-week-old *Ggcx^Δliver/Δliver^* mice, massive subcutaneous bleeding was observed even before death. Dissection of pregnant *Ggcx^Δliver/Δliver^* mice just after death revealed uterine as well as vaginal bleeding. Next, we evaluated the life span of *Ggcx^Δliver/Δliver^* mice by keeping them separately without mating. Male *Ggcx^Δliver/Δliver^* mice began to die from day 27 after birth, and all *Ggcx^Δliver/Δliver^* male mice died within 80 days after birth (Figure 4). Female *Ggcx^Δliver/Δliver^* mice began to die from day 39 after birth and 7 out of 11 (63.6%) survived longer than 100 days, unless they became pregnant (Figure 4). None of the control heterozygous littermates (*Ggcx^+/Δliver^* mice) died within the 100 days of the observation period. The shorter life span of male *Ggcx^Δliver/Δliver^* mice was statistically significant compared with male heterozygous littermates. The cause of death seemed to be anemia secondary to bleeding, since subcutaneous bleeding was observed in some *Ggcx^Δliver/Δliver^* mice before death. Interestingly, female *Ggcx^Δliver/Δliver^* mice survived significantly longer than male *Ggcx^Δliver/Δliver^* mice.

## Discussion

Mediation of post-transcriptional modification of substrate proteins by Ggcx is one of the major functions of vitamin K. So far, 19 proteins are known to be substrates of Ggcx and are expressed throughout body, indicating various physiological functions of vitamin K.

In the present study, we showed that liver-specific deficiency of Ggcx caused bleeding diathesis and short life span. We consider the massive bleeding in subcutaneous tissue or body cavity is a direct cause of death since we observed massive subcutaneous bleeding in most of the dead mice. It is also possible that local bleeding in vital organs such as brain can cause death due to bleeding diathesis.

Short life span of liver-specific Ggcx-deficient mice in the present study along with the clinical presentation of vitamin K deficiency indicate the relative importance of hepatic coagulation factors among Ggcx substrates. Coagulation factors II, VII, IX, and X are known to be vitamin K dependent. Therefore, we considered the decreased activity of these coagulation factors to be responsible for the Ggcx-deficient phenotype. Interestingly, although the activity of factors II and IX was decreased in *Ggcx^Δliver/Δliver^* mice, they live much longer than those with a systemic lack of Ggcx. Most mice systemically lacking Ggcx die between embryonic day 9.5 and 18, and the few that survive to term die shortly after birth [15]. Among mice in which genes for vitamin K-dependent coagulation factors had been knocked out, factor II-deficient mice [20] and factor X-deficient mice [21] are partial embryonic lethal. In factor II-deficient fetuses, abnormal phenotypes such as pale yolk sac membrane, empty blood vessels, enlarged pericardial sacs, and distended hearts were observed, which appeared from embryonic day 9.5 to 12.5. In factor X-deficient mice, some fetuses began to die of massive bleeding from embryonic day 11.5 to 12.5, but the blood vessels and yolk sacs of these mice were normal. Considering the phenotypes of factor II-deficient and factor X-deficient mice, it can be inferred that the embryonic lethal phenotype of systemic Ggcx-deficient mice is likely due to abnormalities that developed at midgestation. In the present study, we used an albumin promoter to regulate Cre transcription. The albumin promoter is activated around embryonic day 16.5 [18]; therefore, Ggcx exists in the liver of *Ggcx^Δliver/Δliver^* mice until embryonic day 16.5. This will contribute to a difference between liver-specific and systemic Ggcx-deficient mice, the latter lack Ggcx from the beginning of embryogenesis. Another possible reason that contributes to the difference between these Ggcx-deficient mice is incomplete ablation of Ggcx in *Ggcx^Δliver/Δliver^* mice. We detected slight residual GGCX activity in the liver of *Ggcx^Δliver/Δliver^* mice (Figure 2D) and factors II and IX activities were also detectable in the blood of *Ggcx^Δliver/Δliver^* mice (Figure 3A). In another report using Alb-Cre mice, complete ablation of target gene was observed in 2 months after birth [19]. Thus, it is assumed that residual Ggcx activity can also remain for several weeks after birth. These slight residual activities may have been vital for the survival of *Ggcx^Δliver/Δliver^* mice.

Both factor VII-deficient mice [22] and factor IX-deficient mice [23] displayed bleeding diathesis. The factor IX-deficient mice showed swollen extremities and extensive hemorrhagic lesions following mechanical trauma, although they survived for at least several weeks. In contrast, the factor VII-deficient mice survived to term and followed a normal Mendelian inheritance pattern. However, most of them died perinatally owing to intra-abdominal hemorrhage within 24 hours, and the remaining neonates died from intracranial hemorrhage in 24 days. Considering the aggressive bleeding of factor VII-deficient mice, the residual acitivity of Ggcx in *Ggcx^Δliver/Δliver^* mice may contribute to the survival. Furthermore, Ggcx activity before embryonic day 16.5 may have some preventive effect against postnatal bleeding.

In regard to the phenotypes of conditional deficiency of coagulation factors, factor VII-insufficient mice at the 0.7% expression level compared with wild-type mice could survive to adulthood despite displaying severely downregulated overall thrombin production and caridiac fibrosis at a young adult age [24]. Induction of prothrombin ablation in adulthood using Mx1-Cre caused fatal hemorrhagic events particularly in heart and brain [25]. Liver-specific Ggcx-deficient mice in the present study exhibit a longer life span in comparison with that of prothrombin deletion in adult mice, because the amount of coagulation factors in Ggcx-deficient mice are substantially decreased but even sufficient to survive for several weeks after birth. In comparison with factor VII-insufficient mice, however, it is assumed that severe insufficiency of multiple coagulation factors occurred in liver-specific Ggcx-deficient mice simultaneously.

It is intriguing that mice lacking fibrinogen, the final effector of the coagulation cascade, displayed similar phenotypes to those seen in *Ggcx^Δliver/Δliver^* mice [26]. They suffered from spontaneous abdominal hemorrhage, but long term survival was possible. In fibrinogen-deficient mice, pregnant female ones died from vaginal hemorrhage, which was also observed in *Ggcx^Δliver/Δliver^* mice.

In this study, we observed longer life spans of female *Ggcx^Δliver/Δliver^* mice compared with male *Ggcx^Δliver/Δliver^* mice. Notably, this sexual dimorphism of life span was also observed in fibrinogen-deficient mice [26], although the difference was smaller than that of *Ggcx^Δliver/Δliver^* mice. Considering activities of Ggcx in the livers of *Ggcx^Δliver/Δliver^* mice were not significantly different between male and female (Figure 2D), this sexual dimorphism may be owing to the difference in aggressiveness of behavior between males and females. Typically, males are more aggressive than females [27], which causing males more susceptible to injury. Another explanation for the sexual dimorphism of life span is the procoagulant activity of female sex hormone, estrogen. Clinically estrogen administration as oral contraceptives or hormone replacement therapy is known to be associated with higher risk of venous thrombosis [28]. In experiment using rats, estrogen was shown to prevent decline of prothrombin caused by vitamin K deficiency [29]. This report suggests higher concentration of estrogen in female mice may ameliorate bleeding diathesis due to Ggcx deficiency which is pathologically similar with vitamin K deficiency.

Interestingly, we observed higher activities of Ggcx as well as vitamin K-dependent coagulation factors in wild type male mice than in wild type female mice (Figure 2D and 3A). In the study by Wong et al. [30], some of the procoagulant factor activity levels were higher in male mice than in female mice and were growth hormone-dependent. In the study by Kundu et al., factor IX activity might be higher in one-month-aged male mice compared with one-month-aged females [31]. Since growth hormone has been known to contribute to sexual dimorphism in liver protein expression [32], it is assumed that the hormone will also exert difference in Ggcx activity.

In human, two missense mutations of *GGCX* gene were reported to cause hereditary bleeding disorder due to low activity of vitamin K-dependent coagulation factors [33], [34]. In our study about 60% of female *Ggcx^Δliver/Δliver^* mice survived longer than 100 days indicated small portion of residual Ggcx activity detected in the liver of *Ggcx^Δliver/Δliver^* mice is sufficient to survive unless they got injured or pregnant. This is compatible with the clinical observation that patients with decreased carboxylase activity live several years before diagnosis [33], [34].

In the current study, we successfully generated mice exhibiting liver-specific insufficiency of Ggcx activity. Because systemic Ggcx knockout mice do not live long after birth, our animal model enables to show the phenotype of liver-specific Ggcx deficiency for the first time and also will open up the possibility to evaluate in extra-hepatic organ-specific Ggcx activities using Cre recombinase driven by proper organ-specific promoters. Recently, several clinical and epidemiological studies have suggested extrahepatic actions of vitamin K. Its fracture-prevention effect has been proven in clinical studies [35], [36], and vitamin K2 is used as a drug for osteoporosis treatment in several Asian countries. In epidemiological studies, low serum concentration of vitamin K was reported to be correlated with osteoarthritis [37], dementia [38], and coronary artery disease [39], [40]. Moreover, a biosynthetic enzyme of menaquinone, which is an active form of vitamin K, was found to be expressed in extrahepatic organs [41]. These discoveries along with the many Ggcx substrates expressed throughout the body, suggest the extrahepatic function of Ggcx is worth investigating. The *Ggcx*-floxed mice we created in this study would be useful in clarifying vitamin K action in the whole body.
